# *In Planta* Glycan Engineering and Functional Activities of IgE Antibodies

**DOI:** 10.3389/fbioe.2019.00242

**Published:** 2019-09-25

**Authors:** Laura Montero-Morales, Daniel Maresch, Silvia Crescioli, Alexandra Castilho, Kristina M. Ilieva, Silvia Mele, Sophia N. Karagiannis, Friedrich Altmann, Herta Steinkellner

**Affiliations:** ^1^Department of Applied Genetics and Cell Biology, University of Natural Resources and Life Sciences, Vienna, Austria; ^2^Department of Chemistry, University of Natural Resources and Life Sciences, Vienna, Austria; ^3^School of Basic and Medical Biosciences, King's College London, St. John's Institute of Dermatology, Guy's Hospital, London, United Kingdom; ^4^Breast Cancer Now Research Unit, Guy's Cancer Centre, School of Cancer and Pharmaceutical Sciences, King's College London, London, United Kingdom

**Keywords:** IgE, antibodies, glycan engineering, glycosylation, plants

## Abstract

Human immunoglobulin E (IgE) is the most extensively glycosylated antibody isotype so glycans attached to the seven *N*-glycosites (NGS) in its Fab and Fc domains may modulate its functions. However, targeted modification of glycans in multiply glycosylated proteins remains a challenge. Here, we applied an *in vivo* approach that allows the manipulation of IgE *N*-glycans, using a trastuzumab equivalent IgE (HER2-IgE) as a model. Taking advantage of plant inherent features, i.e., synthesis of largely homogeneous complex *N*-glycans and susceptibility to glycan engineering, we generated targeted glycoforms of HER2-IgE largely resembling those found in serum IgE. Plant-derived HER2-IgE exhibited *N*-glycans terminating with GlcNAc, galactose or sialic acid, lacking, or carrying core fucose and xylose. We were able to not only modulate the five NGSs naturally decorated with complex *N*-glycans, but to also induce targeted glycosylation at the usually unoccupied NGS6, thus increasing the overall glycosylation content of HER2-IgE. Recombinant human cell-derived HER2-IgE exhibited large *N*-glycan heterogeneity. All HER2-IgE variants demonstrated glycosylation-independent binding to the target antigen and the high affinity receptor FcεRI, and subsequent similar capacity to trigger mast cell degranulation. In contrast, binding to the low affinity receptor CD23 (FcεRII) was modulated by the glycan profile, with increased binding to IgE variants with glycans terminating with GlcNAc residues. Here we offer an efficient *in planta* approach to generate defined glycoforms on multiply glycosylated IgE, allowing the precise exploration of glycosylation-dependent activities.

## Introduction

Immunoglobulin E (IgE), normally the least abundant antibody class in human serum, is most commonly known for its role in the allergic response. The principal mechanisms of IgE antibodies in allergic diseases are (i) recognizing allergens through their antigen-binding regions (Fab) and (ii) interacting via their Fc regions with their two cell surface receptors to induce the allergic cascade (Gould and Sutton, 2008). Both Fcε receptors and IgE are heavily glycosylated molecules, implying a potential impact of this post-translational modification on their activities. In fact, IgE is the most heavily glycosylated antibody class, with seven *N-*glycosylation sites (NGS) and ~12% of their molecular weight made up of carbohydrates (see a schematic illustration of human IgE; Supplementary Figure 1). The glycosylation of IgE presents peculiarities, like site-specific occupancy (lack of glycans at NGS6) and differential site glycosylation pattern (oligomannose vs. complex *N*-glycans). Serum IgE is heavily sialylated, with significant *N*-glycan diversity between different (patho-) physiological stages (Arnold et al., 2004; Plomp et al., 2014).

While *N*-glycans have been proven to be involved in the modulation of IgG effector functions, relatively little is known about their role on other antibody classes (Jefferis, 2012). Attempts to explore IgE glycosylation have concentrated on investigating its interaction with its two Fcε receptors, as they are key players in the allergic cascade: the high affinity Fcε receptor I (FcεRI), present on mast cells, basophils and eosinophils, and the low affinity CD23 (FcεRII) found, amongst others, on B cells and activated macrophages. Some studies claim that IgE glycosylation is not essential for binding to FcεRI and subsequent Fc receptor-mediated functions (Basu et al., 1993; Young et al., 1995; Henry et al., 2000), while other investigations have contradicted these results (Nettletone and Kochan, 1995; Helm et al., 1998; Björklund et al., 1999; Hunt et al., 2005). Using deglycosylation or genetic glycosite disruption, another study points to the requirement of the C-terminal NGS (carrying oligomannose *N*-glycans) for FcεRI binding and subsequent initiation of the allergic cascade (Shade et al., 2015). Notwithstanding, a major challenge to explore the role of IgE glycosylation in more detail lies in the difficulty to produce this protein with multiple NGS with a targeted glycosylation pattern. IgE recombinantly produced in mammalian cell cultures exhibit a mixture of glycoforms (Shade et al., 2015; Montero-Morales et al., 2017), which makes a controlled manipulation difficult in this system.

In recent years, plants (and in particular the tobacco-related species *Nicotiana benthamiana*) have become an interesting platform for glycoengineering. Paradoxically, their limited endogenous glycosylation repertoire provides an advantage for glycoengineering, as it results in the production of homogeneous *N*-glycans, with secreted proteins typically carrying only one or two glycoforms, i.e., GnGnXF (GlcNAc_2_Man_3_XylFucGlcNAc_2_) and MMXF (Man_3_XylFucGlcNAc_2_). This feature facilitates a controlled manipulation of *N*-glycans. We recently developed an engineering approach by combining stably engineered *N. benthamiana* glycosylation mutants and transient expression modules to both knock-out and knock-in targeted glycosylation genes (recently reviewed in Montero-Morales and Steinkellner, 2018). In this context, it was shown that plants largely tolerate human-like *N*-glycosylation and become capable of synthesizing sialylated and even poly-sialylated structures (Castilho et al., 2010; Kallolimath et al., 2016). Several human proteins have been generated in plants with targeted *N*-glycosylation profiles resembling human structures. Most of them are IgG antibodies with only one NGS. However, engineering of proteins with multiple NGSs is a challenge. For example, attempts to engineer the sialylation of IgM antibodies (with five NGSs) were not very successful, with only low amounts of sialylated forms (24% compared to 60% in the mammalian cell-derived IgMs). In addition, other structures usually not present on IgM, like incompletely processed hybrid glycans, were detected (Loos et al., 2014). Targeted engineering and detailed characterization of glycoproteins with a glycosylation pattern as complex as IgE antibodies has not been shown yet.

Interestingly, although not fully understood, IgE also plays a vital role in the recognition of cancer by the immune system and recent studies point to possible therapeutic applications of monoclonal IgEs in the context of cancer (Karagiannis et al., 2017; Fazekas-Singer et al., 2018). Based on their high affinity for their FcεRI expressed on tumor-resident cells (e.g., tissue mast cells, macrophages) and the lack of inhibitory IgE Fc receptors (present for IgG), IgE antibodies may offer new options over predominant therapeutic IgG molecules (Josephs et al., 2017; Pellizzari et al., 2019). In breast cancer, comparative studies between therapeutic IgG trastuzumab and an engineered trastuzumab IgE antibody recognizing the tumor-antigen HER2 indicated that the IgE counterpart could complement or possibly improve the clinical performance of trastuzumab (Karagiannis et al., 2009).

In this study, we sought to generate IgE antibodies with targeted glycosylation profiles and to examine their functional activities using a trastuzumab equivalent monoclonal IgE antibody recognizing the breast cancer antigen HER2 (HER2-IgE) as a model. Using *N. benthamiana* and applying extensive glycoengineering, we generated IgE variants with identical protein backbones but distinct *N*-glycosylation patterns that mainly differ in their core glycosylation and terminal residues, i.e., *N*-acetylglucosamine (GlcNAc), galactose or sialic acid. In addition, HER2-IgE was produced in human embryonic kidney (Expi293F) cells (Ilieva et al., 2019) and, as observed in our previous investigation (Montero-Morales et al., 2017), exhibited a mixture of 30 glycoforms (most abundantly, core-fucosylated branched glycans terminating with galactose or sialic acid). Using ELISA and cell-based assays, we show that all recombinant IgE variants bind to the HER2 antigen and to the high affinity FcεRI, irrespectively of their glycosylation pattern. Accordingly, FcεRI-dependent degranulation of mast cells is not affected by the glycosylation status of the antibodies. In contrast, HER2-IgE glycoforms terminating with GlcNAc residues exhibit increased binding to the low affinity receptor CD23 when compared to variants with a mixture of sialylated and galactosylated glycans. We do not observe any effect of core fucosylation or xylosylation on the tested activity assays.

## Materials and Methods

### Cell Line Maintenance

The human breast adenocarcinoma cell lines SK-BR-3 (ATCC, HTB-30) and BT-474, which naturally express the HER2 antigen, were grown in Dulbecco's Modified Eagle's Medium (DMEM GlutaMAX™, Thermo Fischer Scientific, USA) supplemented with 10% Fetal Calf Serum (FCS). The HER2-expressing human breast ductal carcinoma cell line ZR-75-30 (ATCC® CRL-1504™), the rat basophilic leukemia mast cell line RBL-SX38 (Wiegand et al., 1996) expressing the human form of the tetrameric (α*βγ*2) FcεRI receptor, and the lymphoblastoid B cell line RPMI 8866, which highly expresses CD23, were grown in Roswell Park Memorial Institute Medium (RPMI GlutaMAX™, Thermo Fischer Scientific) supplemented with 10% FCS. All cells were maintained at 37°C in 5% CO_2_. Human embryonic kidney (HEK) cell-derived Expi293F™ cells (Thermo Fischer Scientific) were grown on a Stuart orbital shaker (model SSL1), at 125 rpm in 8% CO_2_ in serum-free Expi293 expression medium (Thermo Fischer Scientific).

### IgE Expression and Glycoengineering

Cloning and expression of HER2-IgE in HEK cells (Expi293F™ expression system, HER2-IgE_HEK_) were described earlier (Ilieva et al., 2019). The cloning and expression of the isotype control chimeric NIP-IgE_HEK_, against the hapten 5-iodo-4-hydroxy-3-nitrophenyl, were described earlier (Neuberger et al., 1985). Briefly, the coding sequence of HER2-IgE and NIP-IgE were cloned into pVitro1-hygro-mcs expression plasmids and transiently expressed in Expi293™ cells using the ExpiFectamine™ 293 transfection kit (Thermo Fischer Scientific), according to the manufacturer's instructions.

For experiments in *N. benthamiana* plants, the cloning and expression of HER2-IgE (heavy and light chains) and of the *Leishmania major* oligosaccharyltransferase single subunit STT3D (LmSTT3D) were described elsewhere (Montero-Morales et al., 2017; Castilho et al., 2018). In order to modulate the glycosylation profile, HER2-IgE was transiently expressed in different plant host by agro-infiltration: the wild-type *N. benthamiana*, WT; the glycosylation mutant with down-regulation of xylosyl- and fucosyltransferases, ΔXTFT (Strasser et al., 2008); the glycosylation mutant expressing a modified human β1,4-galactosyltransferase, ΔXTFT^Gal^ (Schneider et al., 2015); and the mutant line expressing six mammalian genes necessary for protein sialylation, ΔXTFT^Sia^ (Kallolimath et al., 2016). Agro-infiltration experiments were carried on 4–5-week-old plants using Agrobacteria at optical density (OD_600_) 0.05–0.1. Protein expression was monitored 3–5 days post-infiltration. The procedure was recently described in detail (Loos and Castilho, 2015). The six different IgE variants were termed according to their host and/or glycosylation as HER2-IgE_WT_, HER2-IgE_Δ*XF*_, HER2-IgE_Gal_ HER2-IgE_Sia_, HER2-IgE_OST_ HER2-IgE_HEK_, and NIP-IgE_HEK_. Notably, HER2-IgE_OST_ refers to a pooled sample of HER2-IgE produced in ΔXTFT, ΔXTFT^Gal^, and ΔXTFT^Sia^ plants.

### IgE Purification

HER2-IgE_HEK_ was purified using a HiTrap KappaSelect (GE Healthcare, USA) pre-packed column and NIP-IgE_HEK_ was purified using a CaptureSelect™ LC-lambda (Hu) Affinity Matrix (Thermo Fischer Scientific) pre-packed column. Antibodies were eluted with 0.2 M Glycine/HCl + 0.01% NaN_3_ (pH 2.3), neutralized with 1 M Tris (pH 9) and dialyzed against 1 × PBS overnight. After dialysis, HER2-IgE samples were concentrated with Amicon centrifugal filters, MWCO 10,000 kDa (Merck Millipore, Germany).

For plant-produced HER2-IgE, 100 g of infiltrated leaves were harvested 4 days post-infiltration, flash-frozen in liquid nitrogen and crushed with a mortar and pestle. Total soluble proteins were extracted in 1.5 M NaCl, 45 mM Tris, 1 mM EDTA, and 40 mM ascorbic acid for 1.5 h at 4°C (2:1 buffer/fresh leave weight). The extract was then centrifuged on a Sorvall® RC 6™ Plus Superspeed Centrifuge (Thermo Scientific) using a SLA-1500 Rotor at 31,916 *g* for 20 min, filtered through Miracloth and clarified with pH precipitation (bring pH down to 5 for 5 min, then bring up to 7). The supernatant was centrifuged for 20 min using an SS-34 rotor at 47,808 *g*, followed by successive filtration steps through an 8–12 μm filter and a 2–3 μm filter. These centrifugations and filtration steps were repeated until a clear extract was obtained. Using an ÄKTA pure chromatography system (GE Healthcare), a Protein A column (Protein A Sepharose™ Fast Flow, GE Healthcare) was washed with ddH_2_O and equilibrated with 1 × PBS. Then, the clarified total soluble protein was applied to the column at a flow rate of 2 ml/min. The column was washed with 20 CV 1 × PBS and antibodies were eluted with 0.1 M Glycine/HCl (pH 2.7), immediately neutralized with 1 M Tris (pH 9) and dialyzed against 1 × PBS overnight.

All IgE antibodies were subjected to preparative size exclusion chromatography (SEC) using a HiLoad 16/600 Superdex 200 prep grade column (GE Healthcare) in order to separate monomeric IgE from aggregates, degradation products, and other impurities. The column was equilibrated with 1.5 CV running buffer (1 × PBS, 200 mM NaCl, pH 7.4) before loading the sample. All steps took place at a flow rate of 0.8 mL/min. The fractions corresponding to the monomeric peak were collected and concentrated with Amicon centrifugal filters, MWCO 10,000 kDa (Merck Millipore).

### Size Exclusion Chromatography—Multi-angle Light Scattering (SEC-MALS)

HPLC-SEC combined with multi-angle light scattering (SEC-MALS) was performed to confirm the molar mass of the monomeric IgE resulting from preparative SEC. HPLC (Shimadzu prominence LC20, Japan) was equipped with MALS (WYATT Heleos Dawn8+ plus QELS; software ASTRA 6), refractive index detector (RID-10A, Shimadzu) and a diode array detector (SPD-M20A, Shimadzu). The column (Superdex 200 10/300 GL, GE Healthcare) was equilibrated with running buffer (1 × PBS, 200 mM NaCl, pH 7.4). Prior to analysis, the IgE sample was centrifuged (17,000 *g*, 10 min, 20°C) and filtered (0.1 mm Ultrafree-MC filter, Merck Millipore). Experiments were performed at a flow rate of 0.75 mL/min at 25°C. The proper performance of molar mass calculation by MALS was verified by the determination of a sample of bovine serum albumin.

### Glycan Analysis

For the glycan analysis of HER2-IgE, we followed the liquid chromatography-electrospray ionization-tandem mass spectrometry (LC-ESI-MS/MS)-based protocol described in Montero-Morales et al. (2017). Briefly, the heavy chain band were excised from an SDS-PAGE, S-alkylated with iodoacetamide and proteolytically digested with different proteases or combinations of them to generate glycopeptides that cover all *N-*glycosites (NGS): NGS1 (Asn21): NIPSNATSVTL; NGS2 (Asn49): DTGSLNGTTM; NGS3 (Asn99): VAHTPSSTDWVDNK; NGS4 (Asn146): TINIT; NGS5 (Asn252): GTVNLTW; NGS6 (Asn264): ASGKPVNHSTR, and NGS7 (Asn275): NGTLTVTSTLPVGTR. NGS1 and 2 were obtained by the combined trypsin and chymotrypsin digestion; NGS3, 5, 6, and 7 are tryptic peptides, and NGS4 was obtained by digestion with proteinase K. Following appropriate treatment, the peptide mixture was analyzed using a Dionex Ultimate 3000 system directly linked to a Q-TOF instrument (maXis 4G ETD, Bruker) equipped with the standard ESI source (end plate offset 500 V; capillary 4,500 V; dry gas (nitrogen) 5.0 L/min; dry temp 200°C) in the positive ion, data dependent acquisition mode. MS-scans were recorded (range: 150–2,200 m/z, spectra rate: 0.5 Hz). Instrument calibration was performed using an ESI calibration mixture (Agilent). For peptide separation, a Thermo BioBasic C18 separation column (5 μm particle size, 150 × 0.32 mm) was used. For the relative quantification of the different glycoforms, peak areas of EIC (Extracted Ion Chromatograms) of the first four isotopic peaks were summed. All observed charge states and ammonium adducts, as well as formylated glycopeptides, were considered. MS/MS spectra were used for the verification of the glycopeptides by detection of oxonium ions HexNAc (m/z = 204.1), Hex+HexNAc (m/z = 366.1) and the unique Y1 ion (peptide + HexNAc).

*N*-glycosylation site occupancy was calculated from the ratio of deamidated to unmodified peptide upon N-glycan release with 0.15 mU of PNGase A (Europa Bioproducts) overnight at 37°C.

### ELISA

Medium-binding microtiter plates were coated with the extracellular domain of HER2 (HER2-ECD, 69 kDa, produced in-house in CHO Lec1 cells). 1 μg/mL HER2-ECD was diluted in 0.5 M carbonate buffer and the plates were coated overnight at 4°C. Control wells were coated with 1% BSA in 0.5 M carbonate buffer. The plate was washed with 200 μL 0.05% Tween-20 in 1 × PBS (washing buffer) three times, gently shaking for 5 min each time. Blocking was performed with 200 μL washing buffer supplemented with 2% BSA (blocking buffer) for 1 h at room temperature, gently shaking. Blocking buffer was subsequently removed and serially diluted IgE-HER2 antibody (1:2 series dilutions, concentrations ranging between 2.44 and 5,000 ng/mL) were added on the plate, in triplicate, and incubated for 1 h at room temperature, gently shaking. Following three 5 min washes, 100 μL of HRP-conjugated anti-human kappa light chain antibody (A7164, Merck Millipore) diluted 1:5,000 in blocking buffer were added to each well and incubated for 1.5 h at room temperature, gently shaking. After three 5 min washes, 100 μL HRP substrate (3,3′,5,5′-TMB, T0440 Sigma) were added to each well and incubated for 15 min in the dark. Subsequently, 100 μL 2 M H_2_SO_4_ were added to stop the coloring reaction. Absorbance was read at 450 nm, 1 s. The values from eight BSA-coated wells as well as two HER2-ECD-coated wells with no antibody and two HER2-ECD-coated wells incubated with unspecific antibody were subtracted from the mean values of the three technical repeats of each sample. Data was analyzed on GraphPad Prism 7 and is presented as mean values with error bar indicating the standard deviation. EC_50_ was calculated using a four-parameter variable slope regression.

### Flow Cytometric Evaluations of Antibody Binding to Cell Surface Receptors

Antibody binding to the membrane-bound tumor-associated antigen HER2 and to the FcεRI and trimeric CD23 receptors was analyzed using a flow cytometric approach. HER2-expressing and FcεRI-expressing adherent cells were detached using 0.25% trypsin-EDTA, resuspended in FACS buffer (1 × PBS, 2% FCS) (10^6^ cells/ml) and incubated on ice with serially diluted HER2-IgE antibody variants (1:3 dilutions, concentrations ranging between 0.011 and 25 μg/mL), or with the isotype negative control NIP-IgE_HEK_ (used for HER2-expressing cell lines), for 30 min in 96-well round-bottom plates (100 μL, 10^5^ cells per well). Cells were subsequently washed with 1 × PBS (spinning at 500 *g* for 5 min) and incubated on ice for 20 min with either anti-heavy chain detection antibody, namely goat anti-human IgE–fluorescein isothiocyanate (FI-3040; Vector Laboratories) (1.5 μg antibody per well, diluted in 50 μL 1 × PBS). This was followed by another wash with 1 × PBS and final resuspension of the cells in 200 μL FACS buffer. The protocol was adapted for analyzing CD23 binding on RPMI8866 cells: 5 × 10^4^ suspension cells were used per well (instead of 10^5^) and were resuspended in RPMI medium + 2% FCS in place of FACS buffer, in order to have a higher Ca^2+^ concentration. The antibodies were also diluted in RPMI medium + 2% FCS. Samples were analyzed on a BD LSRFortessa™ (BD Biosciences, USA), using the High Throughput Sampler (HTS) option. The results were analyzed using FlowJo software 7.6.5. Data was analyzed on GraphPad Prism 7. The trend line was calculated using a four-parameter variable slope regression.

### Competition Binding

IgE produced in Expi293F cells was labeled with AlexaFluor647 (Thermo Fisher Scientific) according to manufacturer's instructions. RPMI8866 cells were harvested and resuspended in RPMI + 2% FBS. For every sample, 5 × 10^4^ cells were incubated with the labeled AlexaFluor647-Expi293F IgE (25 μg/mL) and the unlabeled IgE glycovariant (0, 4, 2, 1, 0.5 times the labeled IgE), in a final volume of 100 μL. Samples were incubated for 30 min at 4°C followed by a wash with 3 mL of RPMI + 2% FBS. Samples were resuspended in RPMI + 2% FBS and acquired at FACS Canto II (BD Biosciences). The results were analyzed using FlowJo software 7.6.5 and GraphPad Prism 7.

### Degranulation Assays

We evaluated the ability of HER2-IgE variants to trigger degranulation *in vitro* using the rat basophilic mast cell line RBL-SX38, which expresses the human FcεRI receptor as a α*βγ*2 tetramer, the form naturally expressed on the surface of human mast cells. 10^4^ RBL-SX38 cells in 100 μL DMEM + 10% FCS were plated in each well of a 96 well flat-bottomed tissue culture plate and incubated overnight at 37°C in a humidified CO_2_ incubator. The following day, cells were sensitized with the different HER2-IgE variants (and the isotype NIP-IgE_HEK_) diluted in culture medium at 400 ng/mL, incubated for 2 h at 37°C and washed twice with Hanks' Balanced Salt solution (HBSS) + 1% FCS (wash buffer). The negative control for the sensitization step was the incubation with medium (without HER2-IgE).

IgE crosslinking and subsequent triggering of cell degranulation were achieved by incubating the wells for 45 min with either 100 μL of polyclonal rabbit anti-human IgE (Dako) (final concentration: 1.5 μg/mL) or HER2-expressing SK-BR-3 cells (3 × 10^4^ cells/well) in wash buffer at 37°C. The negative control treatment for the degranulation step was wash buffer (no crosslinker). The positive control for degranulation was wash buffer +0.1% Triton-X-100 (Tx) for total degranulation.

Degranulation was terminated by placing the cells on ice and it was measured by quantification of β-hexosaminidase release, assayed using a fluorogenic substrate (4-methylumbelliferyl-N-acetyl-β-D-glucosaminide) prepared according to a standard protocol (1 mmol/L 4-methylumbelliferyl N-acetyl-b-D-glucosaminide, 0.1% dimethyl sulfoxide, 0.1% Triton X-100, 200 mmol/L citrate, pH 4.5 (Linko-Lopponen and Makinen, 1985). Twenty-five microliter of culture supernatant were transferred to a FluoroNunc™ black 96 well plate and diluted with 25 μL wash buffer. Fifty microliter fluorogenic substrate were added, the plate was sealed and incubated at 37°C for 2 h in the dark. The reaction was then quenched with 100 μL/well of 0.5 M Tris pH 8.2 and the plate was read with a FLUOstar Omega Microplate Reader (350-nm excitation and 450-nm emission; BMG Labtech). All measurements were made in triplicate for each condition and degranulation was normalized between 100% degranulation (treatment with 0.1% Triton-X-100 in wash buffer) and 0% (background release from cells sensitized with medium alone and treated with wash buffer alone).

## Results

### Expression of HER2-IgE in *Nicotiana benthamiana* and Expi293F Cells

Recently we reported the expression of an engineered trastuzumab IgE antibody recognizing the tumor-antigen HER2 (HER2-IgE) in Expi293F cells (HER2-IgE_HEK_) and in *N. benthamiana* plants (Montero-Morales et al., 2017). Here, HER2-IgE was transiently expressed in *N. benthamiana* with altered genetic backgrounds: wild type, WT; ΔXTFT, a glycosylation mutant lacking the plant-specific core *N*-glycan residues β1,2-xylose and α1,3-fucose (Strasser et al., 2008); ΔXTFT^Gal^, a glycosylation mutant that elongates GnGn glycans with β1,4-galactose (Schneider et al., 2015); and ΔXTFT^Sia^, a glycosylation mutant for human-type sialylation (Kallolimath et al., 2016). Additionally, to increase NGS occupancy (and induce the glycosylation of the normally unglycosylated NGS6), we co-expressed HER2-IgE with the STT3D oligosaccharyltransferase (OST) from the protozoan *Leishmania major* in ΔXTFT, ΔXTFT^Gal^ and ΔXTFT^Sia^ plants. OST has been used previously to increase NGS occupancies of recombinant human proteins expressed in plants (Castilho et al., 2018). HER2-IgE was also produced in the Expi293™ Expression System (Thermo Fischer Scientific), as recently described (Ilieva et al., 2019).

**Figure 1:**
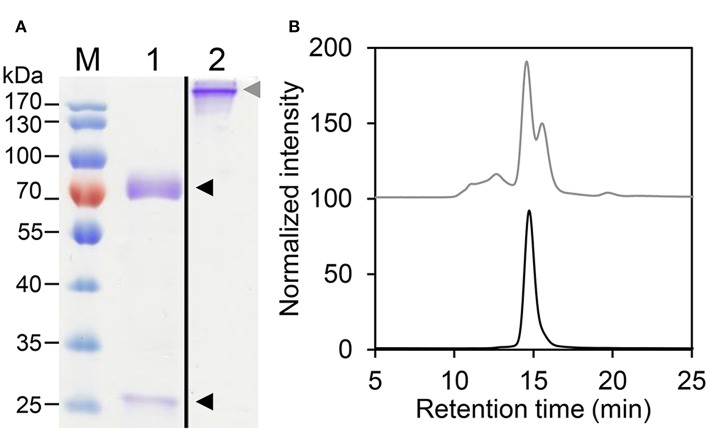
Biochemical characterization of HER2-IgE. **(A)** Coomassie brilliant blue stained SDS-PAGE of purified plant-produced HER2-IgE (4 μg). M, molecular weight marker; 1, reducing conditions; 2, non-reducing conditions. Black arrowheads: heavy and light chains; gray arrowhead: assembled HER2-IgE. Molecular weight shown in kilo Dalton (kDa). **(B)** Size Exclusion-HPLC measurements of HER2-IgE_ΔXF_ before (gray) and after (black) preparative SEC.

The different HER2-IgE variants (Supplementary Table 1) were purified by immunoaffinity chromatography followed by size exclusion chromatography (SEC) to isolate monomeric IgE. HER2-IgE variants were monitored on SDS-PAGE analysis under reducing and non-reducing conditions. The results show the presence of heavy and light chains (75 and 25 kDa, respectively) and fully assembled IgE (190 kDa, Figure 1A). Correct assembling was further confirmed by SE-MALS (Figure 1B).

### Site-Specific *N*-Glycosylation Occupancy and Profiling of HER2-IgE

**Figure 2:**
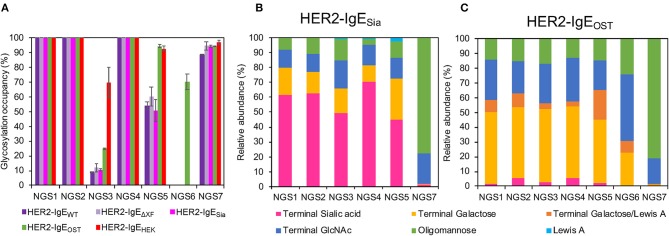
Analysis of the glycosylation status of HER2-IgE. **(A)** *N*-glycosylation site (NGS)-specific occupancy (%). Analysis of NGS1-7 of HER2-IgE expressed in *Nicotiana benthamiana* wild type plants (HER2-IgE_WT_), glycosylation mutants ΔXTFT (HER2-IgE_ΔXF_), ΔXTFT^Sia^ (HER2-IgE_Sia_), co-expressed with *L. major* STT3D oligosaccharyltransferase (OST) in any *N. benthamiana* line (HER2-IgE_OST_), and expressed in human Expi293F cells (HER2-IgE_HEK_). The ratio of deamidated to unmodified peptide was calculated for each NGS following N-glycan release with PNGase A treatment. *N* = 2, error bars show the standard deviation. See Supplementary Table 2 for details. **(B)** Relative abundance (%) of glycoforms present in each occupied NGS of HER2-IgE_Sia_. For detailed information, see Supplementary Table 7. **(C)** Relative abundance (%) of glycoforms present in each occupied NGS of HER2-IgE_OST_. For detailed information, see Supplementary Table 8.

The heavy chain of human IgE antibodies carries seven potential *N*-glycosylation sites, here numbered NGS1-7 (see Supplementary Figure 1). First, NGS occupancy rates were monitored by liquid chromatography-electrospray ionization-tandem mass spectrometry (LC-ESI-MS/MS). In HER2-IgE_WT_, HER2-IgE_Δ*XF*_, and IgE-HER2_Sia_, NGS1, NGS2, and NGS4 are 100% occupied; NGS3, 10%; NGS5, 55%; and NGS7, 94% (Figure 2A; Supplementary Table 2). NGS6 is not glycosylated. Co-expression of OST in the different plant hosts significantly improved glycosylation of NGS3 and NGS5; this variant shows 25% occupancy at NGS3 and up to 94% at NGS5. Notably, we obtained up to 70% glycosylation at NGS6. In HER2-IgE_HEK_, NGS1, NGS2, and NGS4 are fully occupied; NGS3, NGS5, and NGS7 exhibit 70, 91, and 97% occupancy, respectively. NGS6 is not glycosylated. These results are in accordance with previous investigations (Montero-Morales et al., 2017).

We have previously reported on the relative abundance of specific *N*-glycans on each NGS of HER2-IgE_HEK_ and plant-derived HER2-IgE_WT_ and HER2-IgE_Δ*XF*_ (Montero-Morales et al., 2017). In short, NGS1-5 of HER2-IgE_WT_ (Supplementary Figure 2, Supplementary Table 3) and HER2-IgE_Δ*XF*_ (Supplementary Figure 3, Supplementary Table 4) predominantly carry a single complex *N*-glycan terminating with GlcNAc with or without core-modifications, depending on the expression host. In contrast, the HER2-IgE_HEK_ glycosylation profile is highly heterogeneous, with more than 30 different complex glycans detected, most of them tri- and tetra-antennary galactosylated or sialylated (Montero-Morales et al., 2017). In common with serum-derived IgE, at NGS7 all recombinantly produced HER2-IgE variants carry oligomannose glycans (Man5-Man9, Man_5_GlcNAc_2_-Man_9_GlcNAc_2_).

While the glycosylation profiles of plant-derived HER2-IgE_WT_ and HER2-IgE_Δ*XF*_ remain similar from batch to batch, we observed differences on the glycans of HER2-IgE_HEK_ derived from different batches. During this study, we compared the glycosylation profiles of two HER2-IgE_HEK_ batches. Core modifications (i.e., fucosylation) and antennarity remain constant, but terminal sugar residues (i.e., galactose and sialic acid) vary significantly (Supplementary Figure 4, Supplementary Table 5). This is particularly pronounced at NGS4, where the 66% galactosylated and 28% sialylated glycans detected in batch 1 contrast with the 8% galactosylated and 85% sialylated in batch 2. Batch 1 (Supplementary Figure 5, Supplementary Table 5) was used in functional assays.

We have also produced IgE variants with *N*-glycans terminating with either with β1,4-galactose or α2,6-sialic acid. Both glycoforms are commonly found in serum IgEs. Our attempts to produce galactosylated HER2-IgE (HER2-IgE_Gal_) in the glycosylation mutant ΔXTFT^Gal^ resulted in heterogeneous profiles with only up to 21% bi-galactosylated (AA) *N*-glycans (Gal_2_GlcNAc_2_Man_3_GlcNAc_2_) (Supplementary Table 6). Due to its heterogeneous glycosylation profile and batch-to-batch inconsistencies, we were not able to generate sufficiently galactosylated HER2-IgE. Conversely, HER2-IgE_Sia_ exhibited efficiently sialylated glycans, with NaNa (NeuAc_2_Gal_2_GlcNAc_2_Man_3_GlcNAc_2_) being the most abundant glycoform. In total, glycans with terminal sialic acid range between 45 and 78%, depending on the NGS (Figure 2B; Supplementary Table 7). In both HER2-IgE_Gal_ and HER2-IgE_Sia_, NGS6 is not occupied and NGS7 carries oligomannose (74–78%) and hybrid structures (21–22%).

Upon co-expression with *L. major* OST, glycosylation profiles of HER2-IgE variants show mainly complex glycans in NGS1-5 and oligomannose structures in NGS7, independently of their expression host (ΔXTFT, ΔXTFT^Gal^, or ΔXTFT^Sia^). Furthermore, the glycans present on NGS6 follow the pattern of NGS1-5. A pooled sample of HER2-IgE produced in ΔXTFT, ΔXTFT^Gal^, and ΔXTFT^Sia^ plants that exhibits significant amounts of β1,4-galactose (over 50%) named HER2-IgE_OST_ (Figure 2C; Supplementary Table 8) was used for functional assays.

### HER2-IgE Antigen Binding

Fab-mediated functions were monitored by the ability of HER2-IgE glycovariants to bind antigen, the extracellular domain of HER2 (HER2-ECD), using an ELISA-based assay. All plant-produced variants bind to the HER2-ECD similarly, with EC_50_ values ranging between 47.3 and 62.5 ng/mL (Supplementary Figure 6, Supplementary Table 9). HER2-IgE_HEK_ also binds to the HER2-ECD, although our results point to slightly lower binding affinity (EC_50_ = 103.9 ng/mL) compared to the plant-produced counterparts.

**Figure 3:**
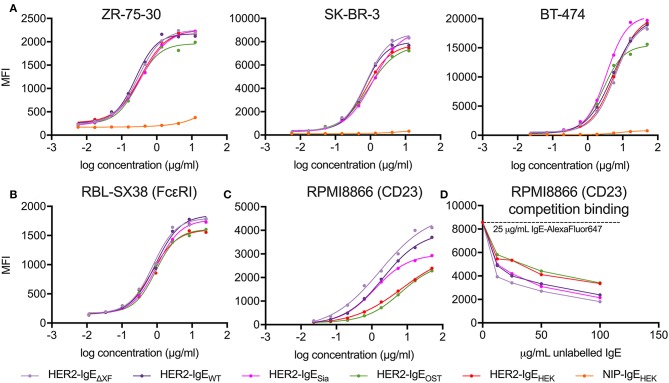
Analysis of antibody target antigen and Fc receptors binding. **(A)** Target antigen recognition of IgE variants on HER2-overexpressing cancer cell lines. All HER2-IgE variants bound to the target antigen in a similar dose-dependent manner in all cell lines used; the negative control (isotype NIP-IgE_HEK_) does not bind. **(B)** Recognition of tetrameric FcεRI on RBL-SX38 cells. All HER2-IgE variants bound to the receptor in a similar dose-dependent manner. **(C)** Recognition of CD23 on RPMI8866 cells. All HER2-IgE variants bound to the receptor in a dose-dependent manner. Differences between the binding behaviors of the variants are observable here. **(D)** Competition binding assay of CD23 on RPMI8866 cells. Graphs are representative of two (in some cases more) independent experiments.

The interaction of HER2-IgE with the target antigen was also assessed by flow cytometry. All HER2-IgE variants recognized HER2 on three different HER2-expressing breast cancer cell lines (ZR-75-30, SK-BR-3 and BT-474) and bound in a dose-dependent manner, with no obvious impact of their glycosylation profiles (Figure 3A). As expected, no binding was observed with the hapten-specific isotype negative control, NIP-IgE_HEK_.

### Fc-Based HER2-IgE Activities

To examine the Fc-mediated properties of HER2-IgE we tested the recognition of Fcε receptors (high affinity FcεRI and low affinity CD23) on human immune effector cells by flow cytometric measurements. Binding of HER2-IgE variants to the high affinity receptor, FcεRI, was studied with the rat basophilic leukemia cell line RBL-SX38, which stably expresses human tetrameric (α*βγ*2) FcεRI. All the antibody variants successfully recognized and bound to the receptor in a dose-dependent manner. A slightly reduced binding was observed in HER2-IgE_OST_ and HER2-IgE_HEK_ samples (Figure 3B).

Binding of HER2-IgE to the low affinity receptor was studied with the human lymphoblastoid cell line RPMI8866, which stably expresses CD23. As with FcεRI, all antibody variants recognized and bound to the receptor. However, we observe an improved binding to CD23 in variants whose glycans terminate with GlcNAc residues (HER2-IgE_WT_ and HER2-IgE_Δ*XF*_) and in those that carry mainly galactosylated structures (HER2-IgE_OST_ and HER2-IgE_HEK_) (Figure 3C). It also should be noted that HER2-IgE_OST_ and HER2-IgE_HEK_ have an overall increased glycosylation content (Figure 2A). HER2-IgE_Sia_ shows similar binding to HER2-IgE_WT_ at low concentrations (0.02–1.85 μg/ml), but at higher concentrations (5–50 μg/ml) its binding is reduced (Figure 3C). These binding differences were also observed in a competition binding assay (Figure 3D).

### Mast Cell Degranulation Assay

The most relevant IgE mediated biological function is the induction of an allergic response. When immunogens crosslink IgE-FcεRI complexes on the cell surface of mast cells, they trigger the early phase of the allergic reaction, which involves mast-cell degranulation: the release of pro-inflammatory mediators such as histamine, tissue matrix-remodeling enzymes and interleukins (Janeway et al., 2001).

**Figure 4:**
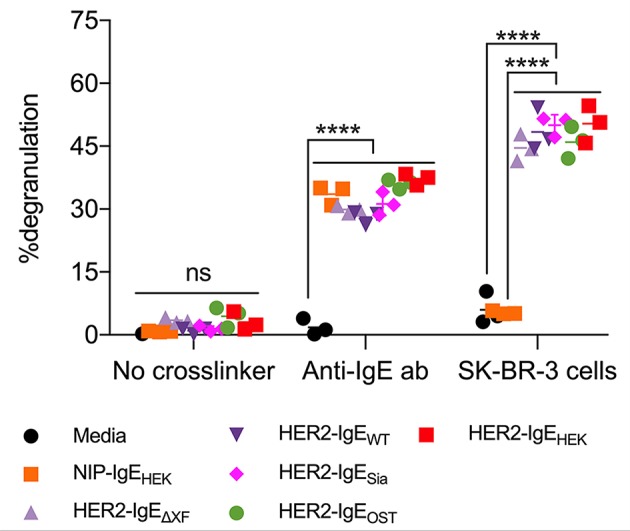
HER2-IgE stimulation of mast cells. IgE-mediated degranulation of RBL-SX38 mast cells measured in negative control (no crosslinker), positive control (polyclonal anti-IgE antibody), and using a HER2-expressing tumor cell line to trigger cross-linking of HER2-IgE-FcεRI complexes. *N* = 3, error bars show the standard deviation. ^****^P < 0.0001; ns, not significant. Data representative of two independent experiments. Isotype NIP-IgE expressed in Expi293F cells (IgE-NIP_HEK_) was used as a control.

Here, we tested the ability of HER2-IgE variants to induce the degranulation of mast cells that express human FcεRI (RBL-SX38 cells). Significant levels of degranulation were detected upon sensitization of mast cells with HER2-IgE followed by crosslinking with either polyclonal anti-IgE or HER2-overexpressing SK-BR-3 breast cancer cells (Figure 4). Sensitization with control NIP-IgE_HEK_ followed by crosslinking with polyclonal anti-IgE also lead to degranulation; importantly, no degranulation was observed with NIP-IgE_HEK_ and SK-BR-3 cells, as NIP-IgE_HEK_ cannot bind to and be cross-linked by HER2-expressing cells. These data, consistent with our findings on FcεRI binding, point to a HER2-IgE glycosylation-independent mast cell degranulation.

## Discussion

In this study, we applied an efficient method for the production of targeted glycoforms of a protein carrying multiple NGS, exemplified by a monoclonal IgE antibody recognizing the breast cancer antigen HER2 (HER2-IgE). HER2-IgE expressed in human Expi293F™ cells exhibited large glycan diversity and batch-to-batch differences, unfavorable factors when studying glycosylation-dependent activities. We took advantage of the limited endogenous glycosylation repertoire of plants, which results in the synthesis of homogeneous complex *N*-glycans. Combining transgenic *N. benthamiana* and transient expression modules, we generate HER2-IgE antibodies glycosylated with human-like complex *N*-glycans at all five NGS that naturally carry complex *N*-glycans.

HER2-IgE_Δ*XF*_ exhibits one dominant glycan, namely GnGn (up to 80%), a structure which can be elongated to generate glycoforms present on serum IgE, i.e., complex *N*-glycans terminating with β1,4-galactose or α2,6-sialic acid. We show the generation of efficiently sialylated HER2-IgE using XTFT^Sia^ plants. The expression of HER2-IgE in XTFT^Gal^ plants in order to generate galactosylated antibodies resulted in low levels of galactosylation and batch-to-batch differences, most probably due to the presence of β-galactosidases (Chandrasekar et al., 2014). By co-expressing OST STT3D from *Leishmania major*, NGS occupancy was substantially increased on the two sites that were inefficiently glycosylated (NGS3 and NGS5). In addition, overexpression of OST initiated glycosylation on NGS6, normally unglycosylated, and glycans at this site were then processed into complex-type, similar to NGS1-5.

IgE antibodies carry three NGSs in the Fab region, so one could expect a link between glycosylation and antigen binding. However, in our studies we do not observe any substantial difference in binding of the HER2-IgE variants to the target antigen on different HER2-overexpressing breast cancer cell lines. Similar to IgG-FcγR interactions, IgE antibodies mediate their pro-inflammatory properties through crosslinking of the FcεRI—IgE complexes on the surface of effector cells (Garman et al., 2000; Sutton et al., 2019). Our results show glycosylation-independent binding of all HER2-IgE variants to FcεRI expressed on the surface of mast cells. Neither terminal sugar residues nor modification of the core sugars resulted in obvious binding differences. Accordingly, degranulation of mast cells, which relies on the interaction and crosslinking of IgE and FcεRI, was not affected by the glycosylation status of the antibodies. These results are in stark contrast to those observed with IgG antibodies, in which binding to FcγRs and subsequent effector functions can be modulated by the glycan composition at the conserved single Fc NGS (Asn297) (Shields et al., 2002; Kaneko et al., 2006; Nimmerjahn et al., 2007). Previously it was shown that NGS7 (Asn275), the proposed equivalent to the single IgG Fc NGS, is crucial for FcεRI IgE binding (Shade et al., 2015). All our IgE glycovariants carry oligomannose structures at NGS7, so we cannot draw conclusions from our study on the role of this NGS on the activities of IgE.

Both IgE and IgG are able to engage a second class of receptors, the evolutionarily related CD23 and dendritic cell-specific intercellular adhesion molecule-3-grabbing non-integrin (DC-SIGN), respectively, which may confer different anti-inflammatory or immune-suppressive responses depending on the immunological context (Anthony et al., 2008; Sutton et al., 2019). These Fc receptors are unique among Ig receptors in that they belong to the C-type lectin-like superfamily (Zelensky and Gready, 2005). However, binding of IgE to CD23 is mediated exclusively through protein–protein interactions, with no known direct carbohydrate interaction (Dhaliwal et al., 2012). Here, in a cell-based assay, we observed that the IgE variants with different terminal sugars have altered CD23 affinities: glycovariants terminating with GlcNAc residues show enhanced binding compared to variants with elongated glycans (Figure 3C). These observations are consistent with data from a competition binding assay, where increased galactosylation and core modifications negatively correlate with CD23 binding (Figure 3D). Currently the effect of terminal sialylation is less clear, since the two samples that carry a substantial portion of terminal sialic acids (HER2-IgE_Sia_ with 44–70% sialylated glycans and HER2-IgE_HEK_ with 26–43%) bind to CD23 differently, with HER2-IgE_Sia_ showing significantly better binding to CD23 than HER2-IgE_HEK_. This could be due to differences in galactosylated glycans, as HER2-IgE_Sia_ has only 9–26% terminal galactose while 47–66% of HER2-IgE_HEK_ glycans are galactosylated. However, we cannot exclude the possible effect of higher NGS occupancy on CD23 binding, since HER2-IgE_OST_ and HER2-IgE_HEK_ have increased NGS occupancy (on NGS3 and NGS5 and, in the case of HER2-IgE_OST_, also on NGS6). *In vitro* studies have proposed that glycosylation of IgG may affect its binding to DC-SIGN and even CD23 (Sondermann et al., 2013; Yu et al., 2013). Our results clearly point to an effect of glycosylation on IgE-CD23 binding. Nevertheless, further studies are needed to clarify structural details.

Collectively, we demonstrate an unmatched glycan engineering approach to generate multiply glycosylated IgE with a targeted glycosylation pattern. Our study opens a door to more precisely exploring of glycosylation-dependent activities, which may be exploited for therapeutic purposes, e.g., in cancer settings or allergic diseases.

## Data Availability Statement

All datasets generated for this study are included in the manuscript/Supplementary Files.

## Author Contributions

LM-M, SC, AC, KI, SM, FA, and HS designed research. LM-M, DM, SC, KI, and SM performed research. LM-M, DM, SC, AC, and KI analyzed data. LM-M, AC, and HS wrote the manuscript. All authors contributed to manuscript revision, read, and approved the submitted version.

### Conflict of Interest

SK is founder and shareholder of IGEM Therapeutics Ltd. The remaining authors declare that the research was conducted in the absence of any commercial or financial relationships that could be construed as a potential conflict of interest.
