# Supplementary material for: In Planta Glycan Engineering and Functional Activities of IgE Antibodies
Source: Front Bioeng Biotechnol. 2019 Sep 25;7:242. doi: 10.3389/fbioe.2019.00242 (PMC6781838; doi:10.3389/fbioe.2019.00242)
Supplement: Supplementary file 1 [file Data_Sheet_1.pdf]

Supplementary Material

Supplementary Material

1 Supplementary Figures and Tables

1.1 Supplementary Figures

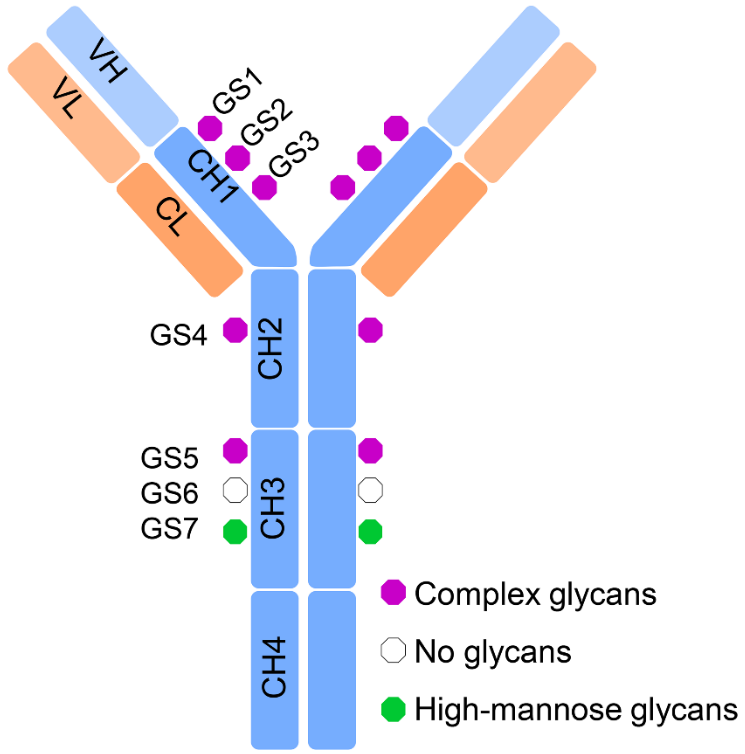

Supplementary Figure 1. Schematic illustration of human serum IgE. Heavy chains (ε chains) shown in blue; light chains shown in orange; immunoglobulin-like constant domains of the heavy chain are numbered (CH1–CH4); variable domains of heavy and light chains (VH, VL). Glycosites (GS) 1–7 on the heavy chain represent Asn21, 49, 99, 146, 252, 264 and 275, and are color-coded according to the glycans found in serum.

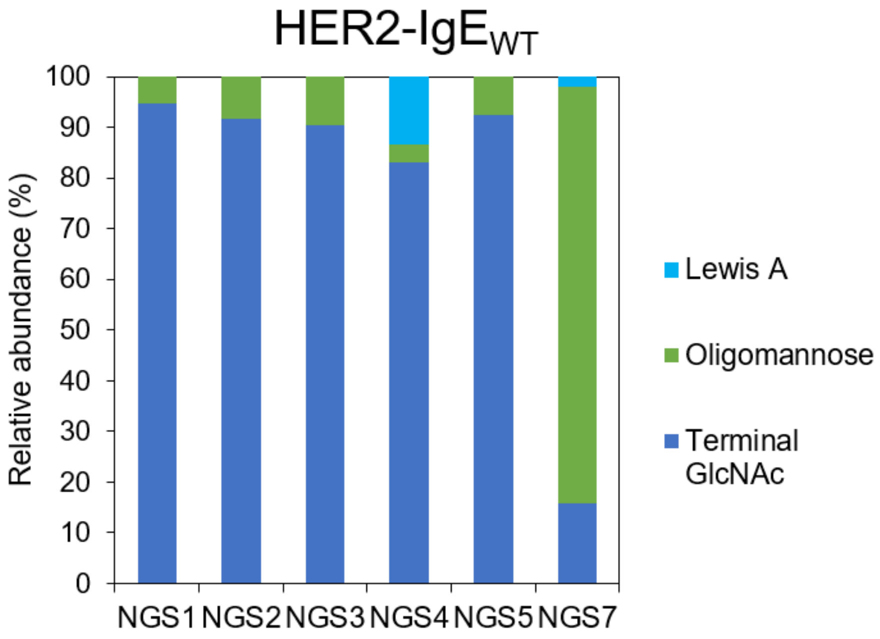

Supplementary Figure 2. Analysis of the glycosylation status of HER2-IgE$_{WT}$. Relative abundance (%) of glycoforms present in each occupied NGS. For detailed information, see Supplementary Table 3.

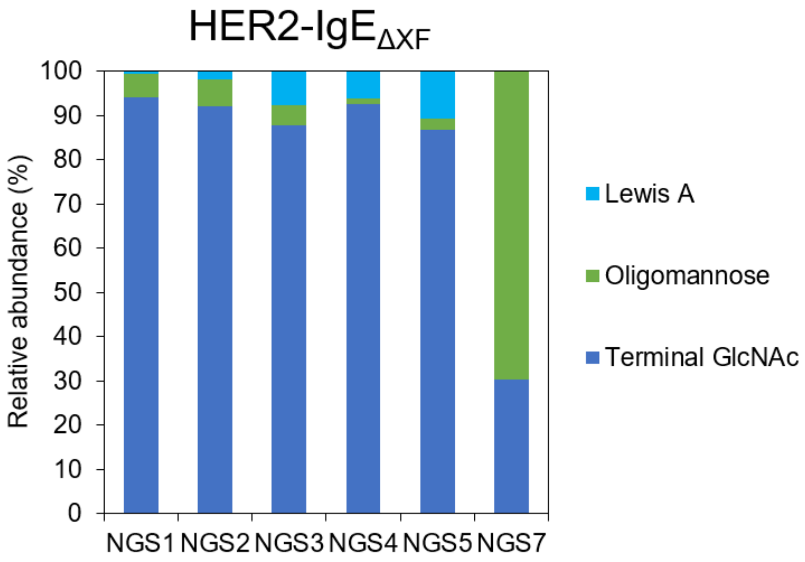

Supplementary Figure 3. Analysis of the glycosylation status of HER2-IgE$_{\Delta XF}$. Relative abundance (%) of glycoforms present in each occupied NGS. For detailed information, see Supplementary Table 4.

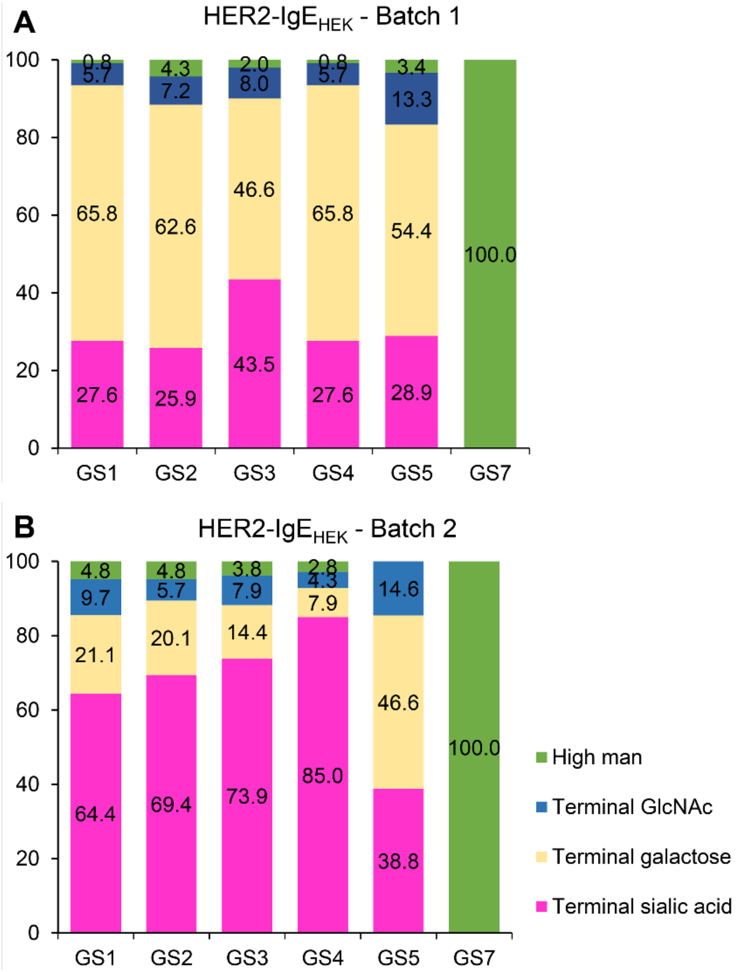

Supplementary Figure 4. Batch to batch glycosylation profiling of HER2-IgE$_{HEK}$. LC-ESI-MS/MS analysis showing the relative abundance (%) of glycoforms present in each NGS of HER2-IgE$_{HEK}$ (A) batch 1: detailed information, see Supplementary Table 5; and (B) batch 2: detailed information see Montero-Morales *et al*, 2017.

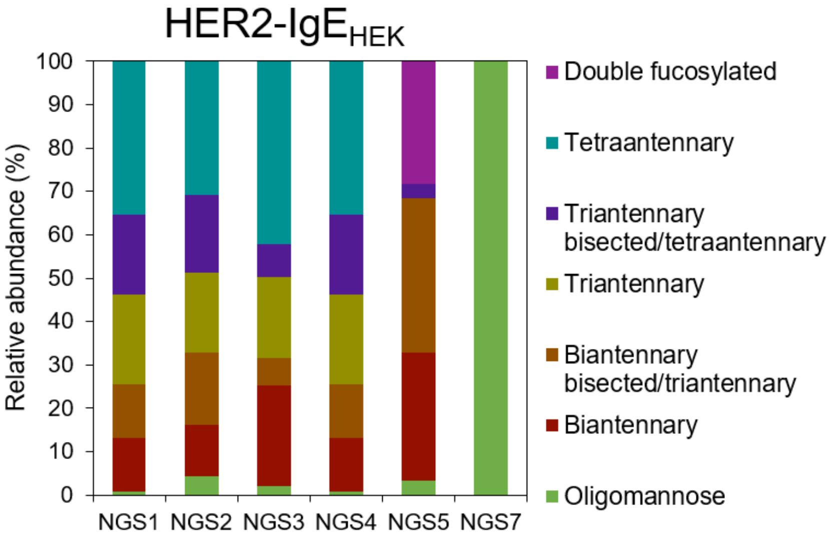

Supplementary Figure 5. Analysis of the glycosylation status of HER2-IgE$_{HEK}$. Relative abundance (%) of glycoforms present in each occupied NGS. For detailed information, see Supplementary Table 5.

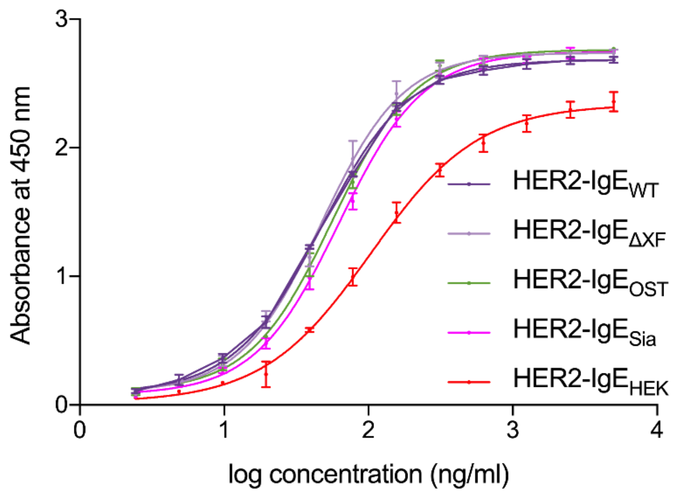

Supplementary Figure 6. Binding to HER2-ECD in an ELISA setting. Data was analyzed on GraphPad Prism 7 and is presented as mean values of three measurements, with error bars indicating the standard deviation. The trend line and EC50 (Supplementary Table 6) were calculated using a four-parameter variable slope regression.

1.2 Supplementary Tables

Supplementary Table 1. IgE variants.

Expressed proteins	Host	Variant name
HER2-IgE	WT	HER2-IgE$_{WT}$
	ΔXTFT	HER2-IgE$_{\Delta XF}$
	ΔXTFT^{Gal}	HER2-IgE$_{Gal}$
	ΔXTFT^{Sia}	HER2-IgE$_{Sia}$
HER2-IgE and OST	ΔXTFT	HER2-IgE$_{OST}$
	ΔXTFT^{Gal}	
	ΔXTFT^{Sia}	
HER2-IgE	Expi293F™ cells	HER2-IgE$_{HEK}$

WT, ΔXTFT, ΔXTFT^{Gal} and ΔXTFT^{Sia} refer to *Nicotiana benthamiana* plants.

Supplementary Table 2. Glycosylation efficiency (%). The efficiency of site-specific glycosylation of HER2-IgE variants was calculated from the ratio of deamidated to unmodified peptide upon N-glycan release with 0.15 mU of PNGase A (Europa Bioproducts) overnight at 37°C. Showed values are the average of two samples ± standard deviation.

Glycosite	HER2-IgE$_{WT}$	HER2-IgE$_{\Delta XF}$	HER2-IgE$_{Sia}$	HER2-IgE$_{OST}$	HER2-IgE$_{HEK}$
NGS1	100.0±0.0	100.0±0.0	100.0±0.0	100.0±0.0	100.0±0.0
NGS2	100.0±0.0	100.0±0.0	100.0±0.0	100.0±0.0	100.0±0.0
NGS3	9.0±0.3	12.0±2.6	10.5±0.9	25.0±0.5	69.6±10.2
NGS4	100.0±0.0	100.0±0.0	100.0±0.0	100.0±0.0	100.0±0.0
NGS5	54.0±2.5	60.3±6.3	50.9±7.5	94.3±1.3	92.6±1.8
NGS6	0.0±0.0	0.0±0.0	0.0±0.0	70.1±5.4	0.0±0.0
NGS7	88.4±0.2	94.6±2.8	94.2±0.9	94.3±0.1	96.9±1.4

Supplementary Table 3. Relative abundance (%) of IgE glycoforms detected in IgE expressed in WT plants (HER2-IgE$_{WT}$). Glycans were analyzed by LC-ESI-MS/MS. Symbol nomenclature in accordance with the Consortium of Functional Glycomics (http://glycomics.scripps.edu/CFGnomenclature.pdf).

Glycoforms			Molecular weight (Da)	NGS1	NGS2	NGS3	NGS4	NGS5	NGS7
Oligomannose structures	Man9+1xHex	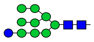	2026.69						1.83
	Man9	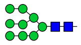	1864.63	1.58	2.71	4.43	0.99	2.57	10.50
	Man8	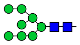	1702.58	2.66	3.27	2.88	1.41	2.51	14.27
	Man7	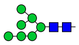	1540.53	1.03	0.92	1.03		2.50	21.06
	Man6	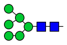	1378.48						15.13
	Man5	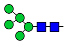	1216.42						18.04
	Man4	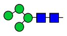	1054.40						1.32
	MMXF	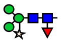	1170.42		1.50	1.15	1.06		
Terminal GlcNAc	GnM	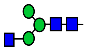	1095.40						1.04
	Man4Gn	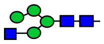	1257.45						2.28
	Man5Gn	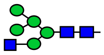	1419.50						3.50
	Man6Gn	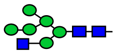	1581.56						0.81
	Man5GnF	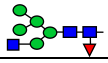	1565.56						2.00
	GnMXF	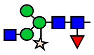	1373.50	4.48	2.86	2.39	2.11	6.24	0.90
	GnGnF	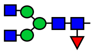	1444.53						3.41
	GnGnXF	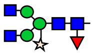	1576.58	90.24	88.73	88.11	81.01	86.18	1.99
Lewis A	MAF	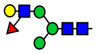	1403.51						1.22
	AGnF	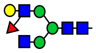	1606.59						0.70
	AGnXF2	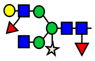	1884.69				6.93		
	AAXF3	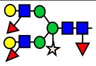	2192.80				6.48		

Mannose GlcNAc Fucose Galactose Xylose

Supplementary Table 4. Relative abundance (%) of IgE glycoforms detected in IgE expressed in ΔXTFT plants (HER2-IgE$_{\Delta XTFT}$). Glycans were analyzed by LC-ESI-MS/MS. Symbol nomenclature in accordance with the Consortium of Functional Glycomics (http://glycomics.scripps.edu/CFGnomenclature.pdf).

Glycoforms			Molecular weight (Da)	NGS 1	NGS 2	NGS 3	NGS 4	NGS 5	NGS 7
Oligomannose structures	Man9+1xHex	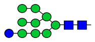	2026.69						0.88
	Man9	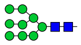	1864.63	1.29	2.12	1.66		0.82	7.38
	Man8	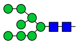	1702.58	2.73	3.16	1.82	0.60	0.88	11.26
	Man7	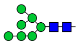	1540.53	0.64	0.67	1.04		0.91	17.77
	Man6	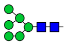	1378.48						13.50
	Man5	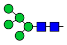	1216.42						16.79
	Man4	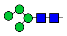	1054.40						1.44
	MM	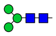	892.32	0.60			0.57		0.74
Terminal GlcNAc	GnM	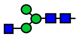	1095.40	2.37	2.05	2.28	2.06	2.56	4.48
	Man4Gn	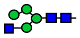	1257.45						9.20
	Man5Gn	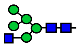	1419.50						14.03
	Man6Gn	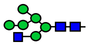	1581.56						0.87
	GnGn	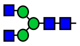	1298.48	79.06	74.97	75.66	82.24	65.78	1.65
	GnGnF	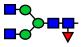	1444.53	12.08	15.14	9.91	8.40	18.46	
	GnMF	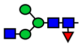	1241.45	0.59					
Lewis A	AGnF	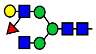	1606.59			5.39	3.68	5.18	
	AGn	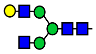		0.62	1.89	2.25	2.46	5.41	

● Mannose ■ GlcNAc ▲ Fucose ● Galactose

Supplementary Table 5. Relative abundance (%) of IgE glycoforms detected in IgE expressed in Expi293F cells (HER2-IgE$_{HEK}$). Glycans were analyzed by LC-ESI-MS/MS. Symbol nomenclature in accordance with the Consortium of Functional Glycomics (http://glycomics.scripps.edu/CFGnomenclature.pdf).

Glycoforms			Molecular weight (Da)	NGS1	NGS2	NGS3	NGS4	NGS5	NGS7
Bi-antennary structures	MGnF	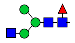	1241.6	0.9	1.9	0.9	0.9	1.6	
	GnGnF	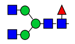	1444.5	2.2		5.0	2.2	5.2	
	GnAF	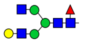	1606.6	2.3	3.1	2.0	2.3	4.7	
	AAF	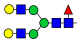	1768.6	4.8	4.7	7.5	4.8	8.5	
	NaGnF	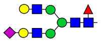	1897.7			1.0		2.4	
	NaAF	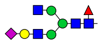	2059.7	2.2	2.2	7.0	2.2	7.0	
Tri-antennary structures not fully galactosylated (or bi-antennary, bisecting GlcNAc)	GnGnGnF	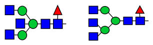	1647.6	2.6	5.3	2.2	2.6	5.3	
	GnGnAF	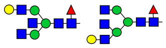	1809.7	2.6	3.9	1.3	2.6	13.1	
	GnAAF	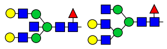	1971.7	4.0	4.5	1.5	4.0	1.4	
	NaGnGnF	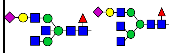	2100.8	0.8	0.6	0.6	0.8	15.7	
	NaAGnF	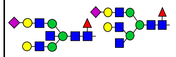	2262.8	2.3	2.4	0.8	2.3		
Tri-antennary structures	AAAF	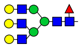	2133.8	15.0	12.3	9.0	15.0		
	NaAAF	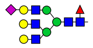	2424.9	5.8	6.2	8.3	5.8		
	NaNaAF	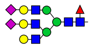	2716.0			1.2			
Tetra-antennary structures<br><br>not fully galactosylated (or tri-antennary, bisecting GlcNAc)	GnGnGnGnF	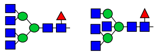	1850.7	1.3	2.8	1.2	1.3	3.2	
	GnGnGnAF	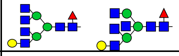	2012.7	1.4	2.3	0.7	1.4		
	GnGnAAF	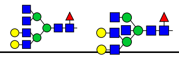	2174.8	3.8	3.6	1.1	3.8		
	GnAAAF	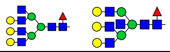	2336.9	6.0	4.9	2.2	6.0		
	NaAGnGnF	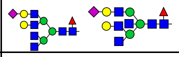	2465.9	1.4	1.0	0.8	1.4		
	NaAAGnF	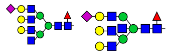	2627.9	4.0	3.3	1.8	4.0		
	NaNaAGnF	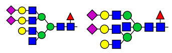	2919.0	0.6	0.0	0.0	0.6		
	AAAAF	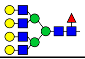	2498.9	24.7	20.7	20.0	24.7		

Tetra-antennary structures	NaAAAF	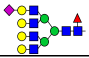	2790.0	9.8	10.2	20.0	9.8		
	NaNaAAF	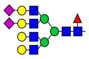	3081.1	0.8	0.0	2.1	0.8		
High-mannose structures	Man5	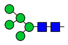	1216.4	0.8	4.3	2.0	0.8	3.4	30.1
	Man6	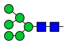	1378.5						26.4
	Man7	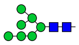	1540.5						26.1
	Man8	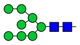	1702.6						13.5
	Man9	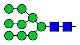	1864.6						3.9
	Man9+1xHex	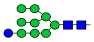	2026.7						
Doubly-fucosylated bi-antennary structures	GnGnF2	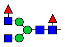	1590.6					1.1	
	GnAF2	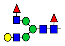	1752.6					2.4	
	GnGnGnF2	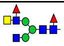	1793.7					2.5	
	GnGnAF2	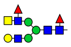	1955.7					14.6	
	GnGnNaF2	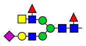	2246.8					3.8	
	GnGnGnGnF2	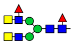	1996.8					4.1	

● Mannose ■ GlcNAc ▲ Fucose ● Galactose ◆ Sialic acid

Supplementary Table 6. Relative abundance (%) of IgE glycoforms detected in HER2-IgE expressed in ΔXTFT plants expressing STGalT (ΔXTFT^{Gal} plants, HER2-IgE$_{Gal}$). Glycans were analyzed by LC-ESI-MS/MS after digestion with trypsin and chymotrypsin (NGS1,2) or trypsin (NGS3,5,7). This sample was not subjected to proteinase K digestion, so NGS4 was not analyzed. Symbol nomenclature in accordance with the Consortium of Functional Glycomics (http://glycomics.scripps.edu/CFGnomenclature.pdf).

Glycoforms			Molecular weight (Da)	NGS1	NGS2	NGS3	NGS5	NGS7
Oligomannose structures	Man9	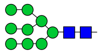	1864.6	4.9		11.4	5.7	8.3
	Man8	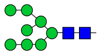	1702.6	6.2	13.7	12.8	10.1	11.8
	Man7	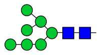	1540.5	5.9	12.1	6.9	6.2	17.5
	Man6	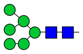	1378.5	1.6		1.3	5.2	15.3
	Man5	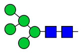	1216.4	2.6	9.2			20.9
Terminal GlcNAc	Man4Gn	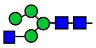	1257.5					9.7
	Man5Gn	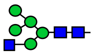	1419.5					12.0
	GnGn	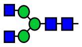	1298.5	40.7	27.5	37.4	25.0	
	GnGnF	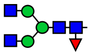	1444.5					1.3
Terminal galactose	AM	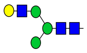	1257.5	15.0	16.9	8.8	25.1	
	AGn	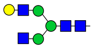	1460.5	6.8		6.2	9.1	
	AA	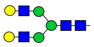	1622.6	16.3	20.6	15.3	13.6	1.8
	AAF	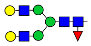	1768.6					1.2

● Mannose ■ GlcNAc ▲ Fucose ○ Galactose

Supplementary Table 7. Relative abundance (%) of IgE glycoforms detected in IgE expressed in ΔXTFT^{Sia} plants (HER2-IgE$_{Sia}$). Glycans were analyzed by LC-ESI-MS/MS. Symbol nomenclature in accordance with the Consortium of Functional Glycomics (http://glycomics.scripps.edu/CFGnomenclature.pdf).

**Glycoforms**			**Molecular weight (Da)**	**NGS 1**	**NGS 2**	**NGS 3**	**NGS 4**	**NGS 5**	**NGS 7**
Oligomannose structures	Man9+1xHex	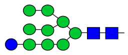	2026.69			1.5			2.1
	Man9	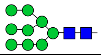	1864.63	2.7	4.8	6.1	2.0	5.0	11.7
	Man8	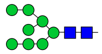	1702.58	3.8	4.5	4.4	1.7	4.3	13.4
	Man7	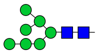	1540.53	1.7	1.3	2.1		1.6	18.7
	Man6	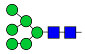	1378.48						14.0
	Man5	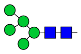	1216.42						16.5
	Man4	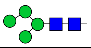	1054.40						1.4
Terminal GlcNAc	GnM	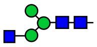	1095.40		1.6	1.8	0.7	1.1	3.2
	Man4Gn	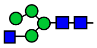	1257.45						7.1
	Man5Gn	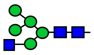	1419.50						7.3
	Man6Gn	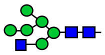	1581.56						2.4
	GnGn	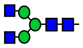	1298.48	12.0	9.3	16.7	12.4	10.9	0.7
	GnGnF	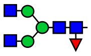	1444.53		1.4	0.7	0.8	1.9	
Lewis A	AGnF	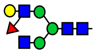	1606.59			1.0	0.8	2.4	
Terminal Galactose	AM	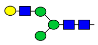	1257.45		0.5	1.4		4.2	
	AGn	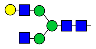	1460.53	1.4	1.1	3.0	2.3	1.9	0.6
	AA	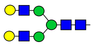	1622.58	11.4	8.0	9.4	6.7	13.2	
	AAF	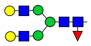	1768.64	5.1	4.7	2.4	1.9	8.7	
Terminal sialic acid	NaM	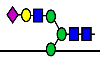	1548.54	7.7	2.7	8.8	5.1	7.7	1.1
	NaGn	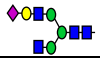	1751.62	3.0	1.2	1.8	2.9	0.8	
	NaA	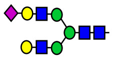	1913.68	8.8	4.5	6.0	5.7	4.5	
	NaNa	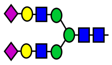	2204.77	36.4	45.5	31.0	53.0	27.1	

	NaMF	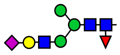	1694.60	0.7	0.7			
	NaAF	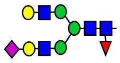	2059.73		0.6		0.7	
	NaNaF	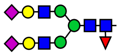	2350.83	5.2	7.6	1.9	3.3	4.7

● Mannose ■ GlcNAc ▲ Fucose ○ Galactose ◆ Sialic acid

Supplementary Table 8. Relative abundance (%) of glycoforms of HER2-IgE co-expressed with OST (LmSTT3D, pooled sample of HER2-IgE produced in ΔXTFT, $\Delta XTFT^{Gal}$ and $\Delta XTFT^{Sia}$ plants). Glycans were analyzed by LC-ESI-MS/MS. Symbol nomenclature in accordance with the Consortium of Functional Glycomics (http://glycomics.scripps.edu/CFGnomenclature.pdf).

**Glycoforms**			**Molecular weight (Da)**	**NGS 1**	**NGS 2**	**NGS 3**	**NGS 4**	**NGS 5**	**NGS 6**	**NGS 7**
Oligomannose structures	Man9+ 1xHex	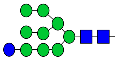	2026.69							1.0
	Man9	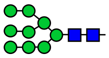	1864.63	2.3	3.6	3.7	1.3	2.7	3.9	8.5
	Man8	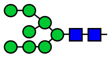	1702.58	4.7	5.7	6.1	2.9	5.3	8.3	12.8
	Man7	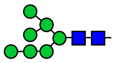	1540.53	2.4	2.2	3.1	1.6	2.1	3.2	15.5
	Man6	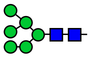	1378.48	1.5	1.4	2.0	1.9	1.4	3.7	14.2
	Man5	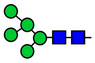	1216.42	1.8	2.2	1.9	2.9	3.6	3.6	17.4
	Man4	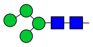	1054.40				1.2			6.3
	MM	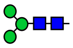	892.32	1.7	0.6		1.2		1.6	5.6
Terminal GlcNAc	GnM	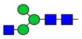	1095.40	8.5	3.0	5.0	2.7	3.9	8.1	0.8
	Man4Gn	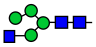	1257.45						21.9	4.1
	Man5Gn	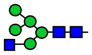	1419.50						3.8	6.7
	Man6Gn	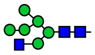	1581.56							5.6
	GnGn	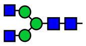	1298.48	18.4	18.7	21.3	25.9	14.1	9.5	
	GnGnF	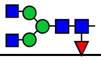	1444.53	0.6		0.5	0.8	2.2	0.9	
	GnMF	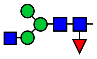	1241.45						0.7	
Terminal Galactose	AM	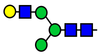	1257.45	11.5	7.4	8.8	5.0	10.5		
	AGn	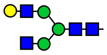	1460.53	5.0	6.0	5.7	6.5	3.0	3.1	
	AA	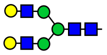	1622.58	31.9	34.7	35.2	36.9	29.6	19.6	0.9
Terminal Galactose/ Lewis A	AMF	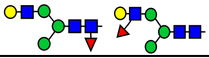	1403.51	0.7	1.3	0.5	0.7	3.3	2.5	
	AGnF	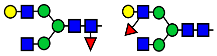	1606.59			0.7	0.8	1.4	1.7	

	AAF	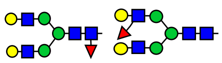	1768.64	7.5	7.8	2.7	2.3	14.8	3.9	0.7
Terminal sialic acid	NaA	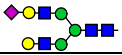	1913.68	1.5	0.0	1.5	1.9	1.2		
	NaNa	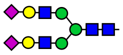	2204.77		5.5	1.3	3.5	0.9		

● Mannose ■ GlcNAc ▲ Fucose ○ Galactose ◆ Sialic acid

Supplementary Table 9. ELISA-derived EC_{50} values, calculated using a four-parameter variable slope regression.

**Variant name**	**EC_{50} (ng/mL)**
HER2-IgE_{WT}	62.47
HER2-$IgE_{\Delta XF}$	47.37
HER2-IgE_{Sia}	47.34
HER2-IgE_{OST}	56.32
HER2-IgE_{HEK}	103.90
